# Assessment of the renal function of patients with anorexia nervosa

**DOI:** 10.1186/s13030-024-00316-6

**Published:** 2024-09-29

**Authors:** Hiroyuki Miyahara, Yoshie Shigeyasu, Chikako Fujii, Chie Tanaka, Mana Hanzawa, Akiko Sugihara, Ayumi Okada, Hirokazu Tsukahara

**Affiliations:** 1 Department of Clinical Pediatrics, Okayama University Academic Field of Medicine, Dentistry, and Pharmaceutical Sciences, 2-5-1 Shikata-Cho, Kita-Ku, Okayama, 700-8558 Japan; 2 Department of Pediatrics, Okayama University Hospital, 2-5-1 Shikata-Cho, Kita-Ku, Okayama, 700-8558 Japan; 3 Clinical Psychology Section, Department of Medical Support, Okayama University Hospital, 2-5-1, Shikata-Cho, Kita-Ku, Okayama, 7008558 Japan; 4 Department of Pediatrics, Dentistry and Pharmaceutical Sciences, Okayama University Graduate School of Medicine, 2-5-1 Shikata-Cho, Kita-Ku, Okayama, 700-8558 Japan

**Keywords:** Anorexia nervosa, Dehydration, Glomerular filtration rate estimated using creatinine, Glomerular filtration rate estimated using cystatin-C, Hypokalemia, Low free triiodothyronine syndrome

## Abstract

**Background:**

A decreased glomerular filtration rate (GFR), estimated using creatinine (Cr– eGFR), is often found at the initial presentation of anorexia nervosa (AN). Its pathophysiology has been explained mainly by dehydration, and chronic hypokalemia is also thought to be a cause. However, because we have often experienced cases of AN with decreased Cr-eGFR without these conditions, we must consider different etiologies. The focus of this paper is on low free triiodothyronine (FT3) syndrome. We also discuss the utility of eGFR, estimated using cystatin-C (CysC-eGFR), for these patients.

**Methods:**

The data of 39 patients diagnosed with AN between January 2005 and December 2023 was available for study. The characteristics of patients at the lowest and highest body mass index standard deviation score (BMI-SDS) were examined. Data on the parameters Cr-eGFR, CysC-eGFR, dehydration markers, potassium (K), and hormonal data and BMI-SDS were assessed during the treatment course to evaluate the correlations in these parameters. Blood hematocrit, uric acid (UA), blood urine nitrogen (BUN) level, and urine specific gravity were adopted as dehydration markers; FT3, free thyroxine, thyroid stimulating hormone, and insulin-like growth factor were adopted as hormonal data. Cr-eGFR and simultaneously evaluated dehydration markers, K, or hormonal data were extracted and correlations associated with the changes in BMI-SDS were examined. Furthermore, Cr-eGFR and simultaneously assessed CysC-eGFR were compared.

**Results:**

When the BMI-SDS was at the lowest value, low-FT3 syndrome was shown. Severe hypokalemia was not found in our study. A linear relation was not found between Cr-eGFR and BMI-SDS. A statistically significant correlation was found between Cr-eGFR and FT3 (*p* = 0.0025). Among the dehydration markers, statistically significant correlations were found between Cr-eGFR and BUN or UA. The difference between Cr-eGFR and CysC-eGFR was prominent, and CysC-eGFR showed much higher values.

**Conclusions:**

Our data indicates that low-FT3 syndrome and dehydration were related to the renal function of our patients with AN. Furthermore, our data suggest that caution is needed in the interpretation of kidney function evaluation when using CysC-eGFR in cases of AN.

## Background

Anorexia nervosa (AN) is an eating disorder that features an intense fear of gaining weight, that commonly occurs in teenage girls, and that is characterized by significant weight loss, amenorrhea, psychological disturbances, and mild hypotension [1]. Compared with constitutionally thin women, AN patients have a more disturbed body image, restricted eating patterns, and higher neuroticism [2]. AN is divided into two subtypes: a restricting type (AN-R) in which a person restricts the amount they eat and a binge-eating/purging type (AN-BP) that is charactered by recurrent episodes of eating large amounts of food in a short time and purging. Purging behaviors in AN-BP are associated with various key factors. AN-BP patients exhibit higher levels of core eating disorder psychopathology, worse global sleep quality, and greater sleep disruption compared with AN-R patients [3, 4]. The severe malnourishment of patients with AN is often accompanied by various physical and psychosocial problems, and careful treatment is required.

Extreme reduction of energy intake is the major cause of physical complications, and every organ can be affected [5]. Impaired kidney function is a frequent complication of AN [6]. In past reports, the renal function of patients with AN has been evaluated mainly by the glomerular filtration rate (GFR), which is estimated using the serum creatinine (Cr-eGFR) level. The cause of decreased Cr-eGFR at the time of diagnosis was often interpreted as dehydration. Chronic hypokalemia has also been discussed as the cause of renal impairment [7, 8]. A case series study of 14 AN patients in Japan found that many had relatively severe and irreversible kidney disease, and the authors hypothesized that chronic hypokalemia was a cause [9]. One case report described end-stage renal failure following a long-term course of AN, suggesting that irreversible renal dysfunction can occur in patients who have the disease long term [10]. In our study, long-term hypokalemia may have contributed to the deterioration of renal function [10]. In experimental models, chronic hypokalemia has been reported to cause ischemia in the kidneys and tubular damage due to the accumulation of ammonium [11]. However, we have often experienced cases of AN with decreased Cr-eGFR without dehydration or hypokalemia, and other etiologies are likely.

Whereas serum creatinine (Cr) is commonly used when evaluating renal function, Cr values are strongly affected by muscle volume; dietary animal protein intake is also a factor influencing the Cr value [12]. Renal function among the patients with AN can be overestimated when Cr-eGFR is used because of a drastic reduction in muscle volume or protein intake. The use of cystatin-C (CysC) has been proposed in some reports [6, 13] for the accurate evaluation of renal function in patients with AN, but sufficient discussion has not yet taken place on whether GFR estimated by CysC (CysC-eGFR) serves as a valid parameter.

Among patients with AN, various endocrine changes occur to adapt to the fasting state [7, 8, 14]. Reduced thyroid function is one that contributes to sustaining life by suppressing metabolism. To adapt to the fasting state, decreased conversion of T4 to T3 occurs (low free tri-iodothyronine (FT3) syndrome), leading to reduced energy expenditure. This reaction can provoke changes in renal function in addition to changes such as bradycardia or hypotension [14–18]. Thus, low FT3 syndrome should be considered when evaluating renal function of patients with AN, but to date there has been insufficient discussion.

It is also known that discrepancy between Cr-eGFR and CysC-eGFR occurs when thyroid function is impaired [19]. While Cr-eGFR decreases, CysC-eGFR is overestimated and does not reflect the actual GFR [19]. In our clinical experience with AN patients, such discrepancies have been frequently observed. Thus, in this study, we focus on the thyroid function of patients with AN and examine the relation between their thyroid and renal function. Furthermore, we also assess CysC-eGFR and Cr-eGFR, and discuss how to evaluate the renal function of patients with AN, especially during low-FT3 syndrome.

## Methods

### Study design and participants

We retrospectively collected data for patients treated in the Department of Pediatrics at Okayama University Hospital, Japan, from January 2005 to December 2023. Inclusion criteria were patients who met Diagnostic and Statistical Manual of Mental Disorders (DSM-5) criteria for AN [20] and who were treated by specialists in psychosomatic medicine for children. In this study, both outpatients and inpatients were included. Exclusion criteria were patients with no available Cr-eGFR or body weight data. Regarding our facility’s treatment policy, we treated patients based on the Japanese Society of Pediatric Psychosomatic Medicine guidelines for the treatment of eating disorders. Patients with physical conditions requiring hospitalization were treated as inpatients, while others were treated as outpatients. Since 2021, family-based treatment (FBT) has been the first choice for outpatients [21].

First, we studied the clinical parameters age, sex, height, weight, body mass index standard deviation score (BMI-SDS), degree of obesity, type of AN, duration of therapy, duration of illness, vital signs (body temperature (BT), heart rate (HR), systolic blood pressure (SBP), diastolic blood pressure (DBP)), physical findings (skin turgor and capillary refill time), and laboratory data. Given the standard weight (stWT (kg)) and actual weight (acWT (kg)), the degree of obesity is calculated using the following formula.

$$\mathbf{degree}\ \mathbf{of}\ \mathbf{obesity} = \left\{(\mathbf{stWT} - \mathbf{acWT})/(\mathbf{stWT})\right\}\times \boldsymbol{100}(\boldsymbol{\%}).$$

When assessing eGFR in Japanese children, the Schwartz formula [22], which has been widely used, has been shown to lack accuracy. Recently, a new formula for estimating eGFR in Japanese children has been reported and is now widely used in Japan. Therefore, we used these estimation formulas to calculate Cr-eGFR or CysC-eGFR [23, 24]. Using the height (HT (m)) value, the reference serum creatinine (ref Cr) for boys and girls was calculated as follows:

$$\mathbf{For}\ \mathbf{boys}: \mathbf{ref}\ \mathbf{Cr}=-\mathbf{1.259}\mathbf{HT}^{\mathbf{5}}+{\mathbf{7.815}\mathbf{HT}}^{\mathbf{4}}-{\mathbf{18.57}\mathbf{HT}}^{\mathbf{3}}+{\mathbf{21.39}\mathbf{HT}}^{\mathbf{2}}-\mathbf{11.71}\mathbf{HT}+\mathbf{2.628}.$$

$$\mathbf{For}\ \mathbf{girls}:\mathbf{ref}\ \mathbf{Cr}=-\mathbf{4.536}\mathbf{HT}^{\mathbf{5}}+\mathbf{27.16}\mathbf{HT}^{\mathbf{4}}-\mathbf{63.47}\mathbf{HT}^{\mathbf{3}}+\mathbf{72.43}\mathbf{HT}^{\mathbf{2}}-\mathbf{40.06}\mathbf{HT}+\mathbf{8.778}.$$

Based on this calculation, the estimated glomerular filtration rate (eGFR) (ml/min/1.73 m^2^) was obtained using the following formula, in which serum creatinine is denoted as s-Cr [23]:

$$\mathbf{Cr}-\mathbf{eGFR}\;=\;\mathbf{110.2}\;\times\;(\mathbf{ref}\;\mathbf{Cr}\;/\;\mathbf{s}-\mathbf{Cr})\;+\;\mathbf{2.93}.$$

CysC-eGFR was calculated as follows [24]:

$$\mathbf{CysC}-\mathbf{eGFR}(\mathbf{mL}/\mathbf{min}/\mathbf{1.73m}^{\mathbf{2}})=\mathbf{104.1}\times\mathbf{1}/\mathbf{serum}\ \mathbf{CysC}(\mathbf{mg}/\mathbf{L})-\mathbf{7.80}.$$

**Fig. 1:**
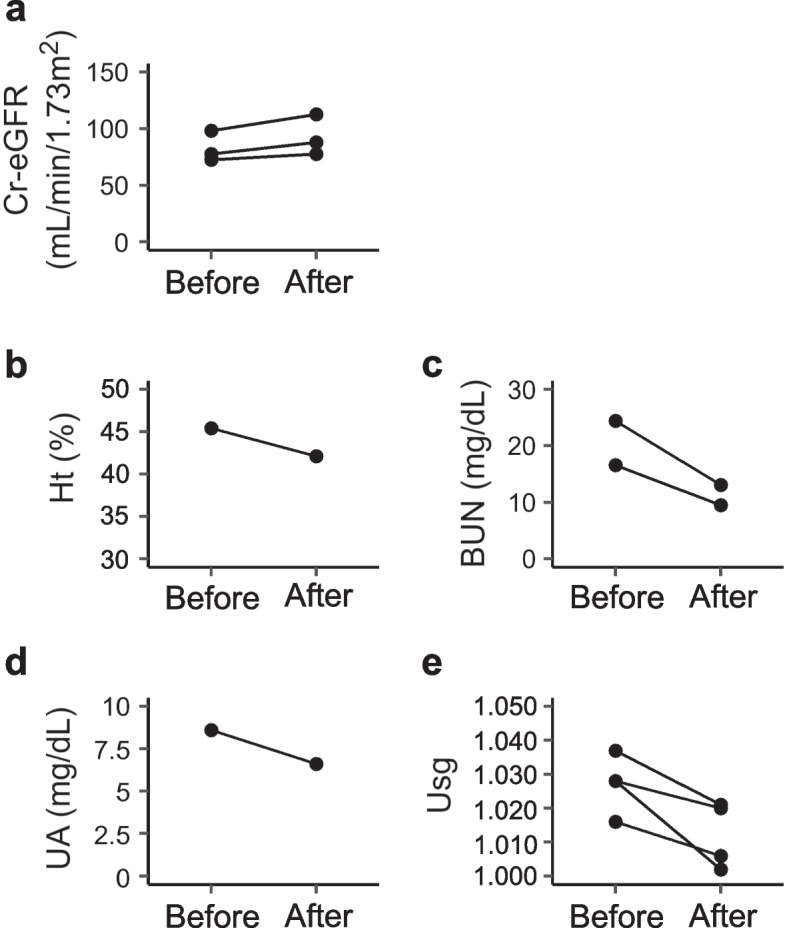
Cases in which dehydration parameters were evaluated before and after fluid infusion at the lowest BMI-SDS. Patients for whom dehydration parameters were evaluated before and after fluid infusion at the lowest BMI-SDS are shown. Additionally, results in which Cr-eGFR was assessed before and after fluid infusion are also presented. For all patients, fluid infusion resulted in (**a**) an increase in Cr-eGFR, and (**b**–**e**) a decrease in dehydration parameters

Using these data, patient characteristics at the time when Cr-eGFR was first measured during the observation period (Table 1) or at the lowest and the highest BMI-SDS were examined (Table 2). Subtypes of AN (AN-R or AN-BP) were defined using DSM-5 criteria. Furthermore, we extracted the data of cases in which infusion therapy was administered at the time of the lowest BMI-SDS, as shown in Table 2, and at least one of the “dehydration markers” was re-evaluated within 3 days. By examining the change in the “dehydration markers”, we investigated responsiveness to infusion therapy (Fig. 1).

**Table 1:** Patient characteristics at the first evaluation of Cr-eGFR

Parameter	N	Median (min–max), or n (%)
Sex, female, n (%)	39	38 (97.4)
Age (years)	39	13 (7–16)
Height-SDS	39	− 1.0 (− 3.0–1.3)
BMI-SDS	39	− 2.0 (− 7.1–1.0)
Degree of obesity	39	− 22.8 (− 47.1–13.3)
Type of AN, AN-R, n (%)	39	37 (94.9)
Duration of illness (months)	25	13 (1–75)
BT (℃)	8	36.3 (35.0–37.0)
HR (bpm)	34	61 (40–100)
SBP (mmHg)	32	94 (62–126)
DBP (mmHg)	32	58 (38–90)
Alb (g/dL)	33	4.7 (3.9–5.5)
Pre-Alb (mg/dL)	28	21 (12–38)
Cr-eGFR (mL/min/1.73 m^2^)	39	96.2 (59.9–126.1)
CysC-eGFR (mL/min/1.73 m^2^)	5	136.8 (127.4–149.9)
Na (mEq/L)	37	140 (135–147)
K (mEq/L)	37	4.2 (3.6–5.3)
Cl (mEq/L)	37	105 (98–111)
Ca (mg/dL)	37	9.4 (8.5–10.2)
P (mg/dL)	36	3.8 (2.5–5.0)
Ht (%)	39	39.4 (33.9–47.3)
UA (mg/dL)	21	4.3 (2.6–6.0)
BUN (mg/dL)	39	16.5 (7.9–42.7)
Usg	29	1.022 (1.003–1.037)
FT3 (pg/mL)	25	1.52 (0.4–3.1)
FT4 (ng/dL)	31	1.04 (0.62–1.47)
TSH (μU/mL)	31	1.08 (0.15–5.58)
IGF1 (ng/mL)	14	64.2 (6.3–284)
ACTH (pg/mL)	3	38.9 (25.8–40.0)
Cortisol (μU/dL)	11	17.8 (8.7–29.8)
LH (mIU/mL)	23	0.3 (0.2–88.9)
FSH (mIU/mL)	23	1.0 (0.3–21.9)
E2 (pg/mL)	22	9.9 (5.0–298.1)

*ACTH* Adrenocorticotropic hormone, *Alb* Albumin, *AN* Anorexia nervosa, *AN-R* Anorexia nervosa restricting type, *BMI* Body mass index, *BT* Body temperature, *BUN* Blood urea nitrogen, *Ca* Calcium, *Cl* Chloride, *Cr* Creatinine, *CysC* Cystatin-C, *DBP* Diastolic blood pressure, *E2* Estrogen, *eGFR* Estimated glomerular filtration rate, *FSH* Follicle stimulating hormone, *FT3* Free triiodothyronine, *FT4* Free thyroxine, *Ht* Blood hematocrit, *IGF1* Insulin-like growth factor, *K* Potassium, *LH* Luteinizing hormone, *max* Maximum, *min* Minimum, *Na* Sodium, *P* Phosphate, *Pre-Alb* Pre-albumin, *SBP* Systolic blood pressure, *SDS* Standard deviation score, *TSH* Thyroid stimulating hormone, *UA* Uric acid, *Usg* Urine specific gravity

**Table 2:** Patient characteristics at the lowest and highest BMI-SDS

Parameter	n	Median (min–max) or n (%)		*p*-value
		**BMI-SDS (min)**	**BMI-SDS (max)**	
Duration of therapy (days)	34	19 (0–3878)	277 (0–1710)	0.073
BMI-SDS	34	− 3.59 (− 8.81– − 0.41)	− 0.39 (− 5.12–1.63)	< 0.001**
Degree of obesity	34	− 30.8 (− 50.6– − 4.52)	− 5.8 (− 38.4–29.7)	< 0.001**
Type of AN	34	32 (94.1)	32 (94.1)	–
BT (℃)	1	36.6	36.3	–
HR (bpm)	25	60 (41–82)	75 (54–112)	< 0.001
SBP (mmHg)	21	85 (72–118)	104 (87–124)	< 0.001
DBP (mmHg)	21	53 (43–77)	62 (46–80)	0.024
Alb (g/dL)	27	4.7 (3.9–5.4)	4.6 (3.8–5.3)	0.11
Pre-Alb (mg/dL)	18	23 (13–36)	29 (20–40)	0.007**
Cr-eGFR (mL/min/1.73 m^2^)	34	84.2 (59.9–130.5)	108.5 (72.4–182.8)	< 0.001**
Na (mEq/L)	32	139 (135–147)	140 (136–142)	0.18
K (mEq/L)	32	4.3 (3.0–4.7)	4.1 (3.7–5.1)	0.73
Cl (mEq/L)	32	105 (90–111)	105 (103–108)	0.24
Ht (%)	34	40.4 (33.9–45.4)	39 (26.5–45.7)	0.096
UA (mg/dL)	34	4.6 (3.5–10.1)	4.4 (3.4–5.9)	0.41
BUN (mg/dL)	34	16.4 (9.7–42.7)	13.3 (6.4–26.2)	< 0.001**
Usg	24	1.016 (1.002–1.039)	1.011 (1.004–1.037)	0.41
FT3 (pg/mL)	4	1.16 (0.83–2.03)	3.57 (2.50–4.06)	–
FT4 (ng/dL)	15	1.01 (0.63–1.44)	1.15 (0.97–1.37)	0.026*
TSH (μU/mL)	15	1.57 (0.09–3.81)	1.39 (0.57–3.53)	0.27
FSH (mIU/mL)	8	1.00 (0.10–4.50)	5.75 (0.30–9.90)	0.022*
E2 (pg/mL)	8	11.2 (5.0–93.0)	30.9 (5.0–167.0)	0.11

*Alb* Albumin, *BMI* Body mass index, *BT* Body temperature, *BUN* Blood urea nitrogen, *Cl* Chloride, *Cr* Creatinine, *DBP* Diastolic blood pressure, *E2* Estrogen, *eGFR* Estimated glomerular filtration rate, *FSH* Follicle stimulating hormone, *FT3* Free triiodothyronine, *FT4* Free thyroxine, *Ht* Blood hematocrit, *K* Potassium, *max* Maximum, *min* Minimum, *Na* Sodium, *Pre-Alb* Pre-albumin, *SBP* Systolic blood pressure, *SDS* Standard deviation score, *TSH* Thyroid stimulating hormone, *UA* Uric acid, *Usg* Urine specific gravity

*p*-values are for Wilcoxon signed-rank test. Statistical analysis was not performed for FT3 because of the small sample size

^*^p < 0.050

**p < 0.01

**Fig. 2:**
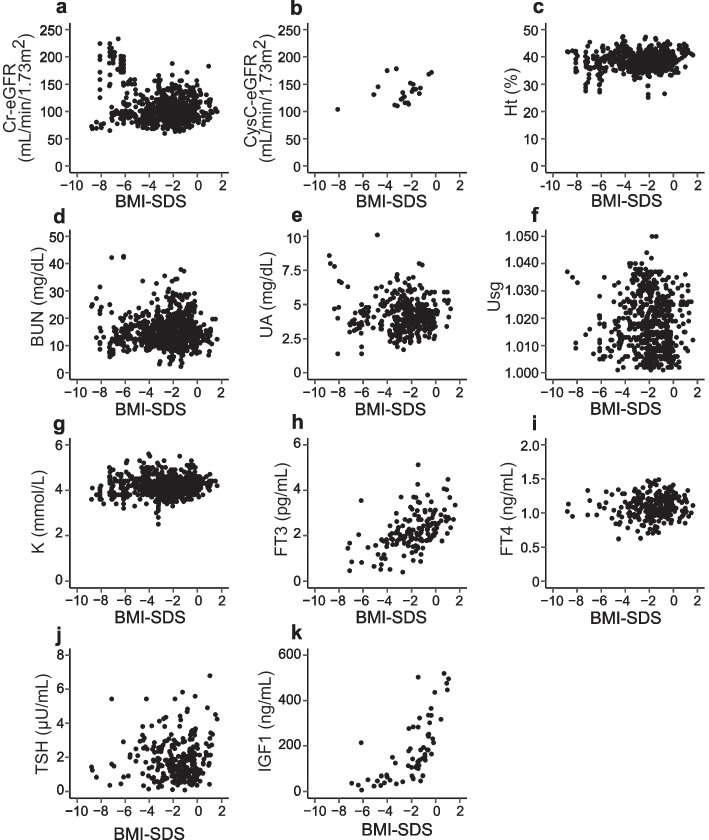
Overall tendency of our patients during the therapeutic process. All results of the relation between each laboratory parameter and BMI-SDS are shown. **a** The Cr-eGFR values showed no linear change. **b** CysC-eGFR showed normal or high values. **c**–**f **The parameters associated with dehydration indicated no obvious tendency, but dehydration was implied in some cases during low BMI-SDS. **g** Overall, serum K was normal and severe hypokalemia was not seen. **h**–**k** For the endocrine biomarkers, the FT3 and IGF1 values seemed to change linearly, even though no obvious tendency was found for TSH or FT4

To capture the overall trends of our patients during the therapeutic process, scatter plots showing the relation between the BMI-SDS and laboratory parameters mentioned above were created (Fig. 2). To create Fig. 2, whole data from all patients and their respective parameters were used.

**Fig. 3:**
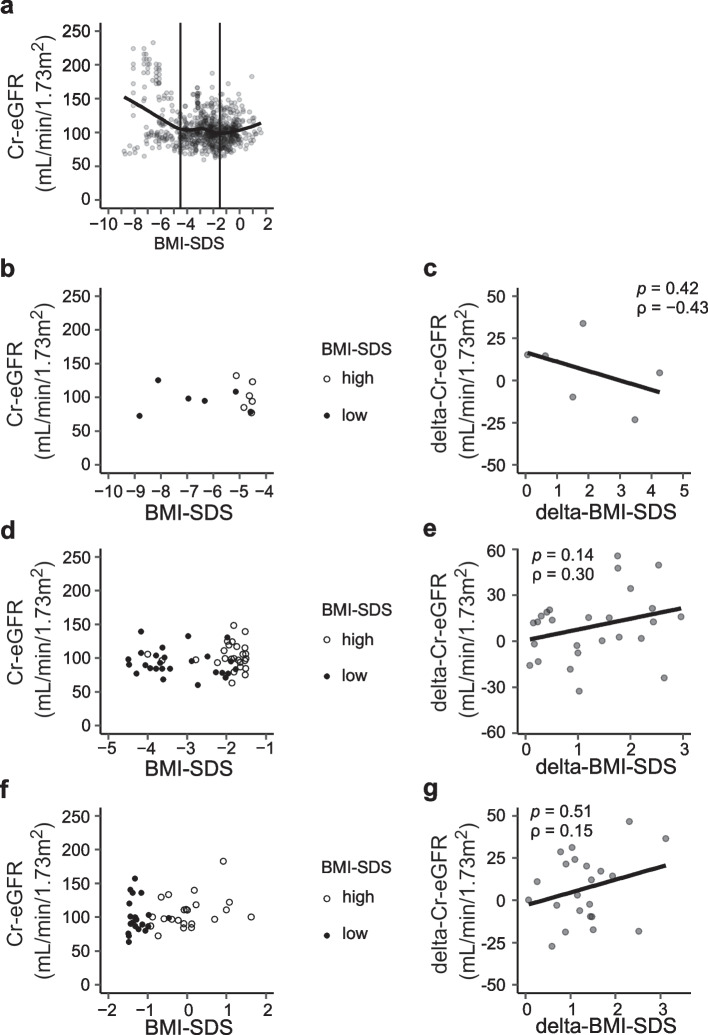
Correlations between Cr-eGFR and BMI-SDS. Relations between Cr-eGFR and BMI-SDS are shown. The LOESS curve shown in (**a**), BMI-SDS was divided into three categories: BMI-SDS ≤  − 4.5 SD, − 4.5 < BMI-SDS ≤  − 1.5 SD, and BMI-SDS >  − 1.5 SD. **b**–**g** Although tendencies for negative correlation during BMI-SDS ≤  − 4.5 SD and positive correlation during − 4.5 < BMI-SDS ≤  − 1.5 SD or BMI-SDS >  − 1.5 SD were found between Cr-eGFR and BMI-SDS, no statistical correlation was demonstrated

The relation between Cr-eGFR and BMI-SDS was further examined. A smooth curve was produced by locally estimated scatterplot smoothing (LOESS) regression in the scatter plots for Cr-eGFR and BMI-SDS, as shown in Fig. 3. BMI-SDS was divided into three categories based upon the LOESS curve: BMI-SDS ≤  − 4.5 SD, − 4.5 < BMI-SDS ≤  − 1.5 SD, and BMI-SDS >  − 1.5 SD. In each BMI-SDS category, the data of patients with at least two data points for BMI-SDS and Cr-eGFR were extracted, and Cr-eGFR values at the lowest and highest BMI-SDS during each category were plotted (Fig. 3). The changes in Cr-eGFR (delta-Cr-eGFR) and BMI-SDS (delta-BMI-SDS) were also plotted, and the correlation of these values was assessed for each BMI-SDS category (Fig. 3).

**Fig. 4:**
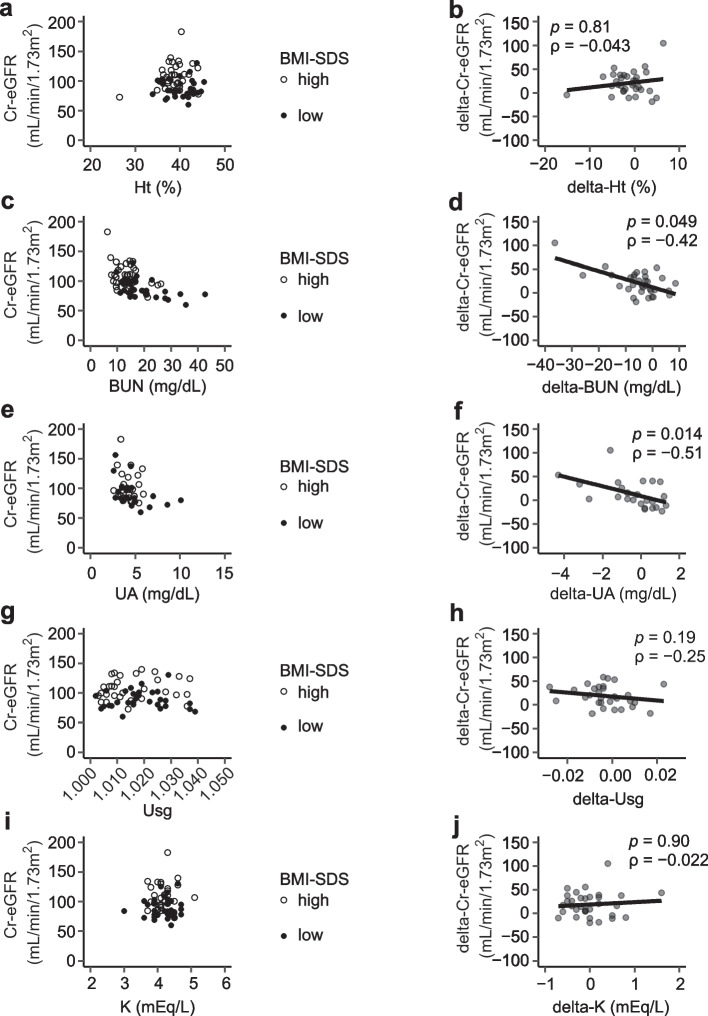
Relation between Cr-eGFR and dehydration parameters or K depending on the changes in BMI-SDS. **a**–**j** The relation between the changes in Cr-eGFR and dehydration parameters or K depending on BMI-SDS in each patient was evaluated. **a**, **c**, **e**, **g**, **i** Cr-eGFR and each parameter at the point of lowest and highest BMI-SDS were drawn as scatter plots. **b**, **d**, **f**, **h**, **j** Statistically negative correlations were found between delta-Cr-eGFR and delta-BUN or delta-UA, but no correlations were found between Cr-eGFR and the other parameters

**Fig. 5:**
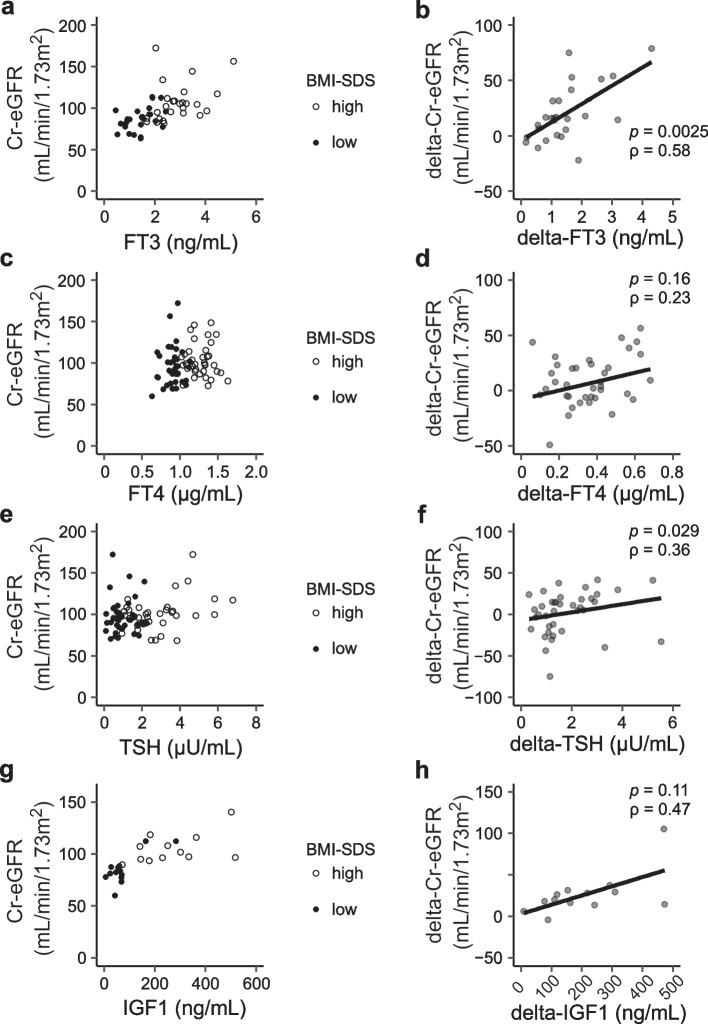
Relation between changes in Cr-eGFR and each hormone depending on the change in BMI-SDS. The relation between changes in Cr-eGFR and hormones depending on BMI-SDS in each patient was evaluated. **a**, **c**, **e**, **g** Cr-eGFR and each hormone at the point of the lowest and highest BMI-SDS were drawn as scatter plots. **b**, **d**, **f**, **h** Statistically significant correlations were found between delta-Cr-eGFR and delta-FT3 or delta-TSH (*p*  = 0.0025, ρ  = 0.58, and *p*  = 0.029, ρ  = 0.36, respectively)

We examined the relation between Cr-eGFR and the dehydration parameters blood hematocrit (Ht), uric acid (UA), blood urea nitrogen (BUN), and urine specific gravity (Usg) or potassium (K) depending on the change of BMI-SDS. The patients were tested for Cr-eGFR and the parameters; in addition, BMI-SDS was analyzed simultaneously at least twice. For these data, values for the respective parameters at the lowest and highest BMI-SDS were selected. Scatter plots were drawn to evaluate the relation between Cr-eGFR and the respective parameters, depending on the change in BMI-SDS. Furthermore, delta-Cr-eGFR and the dehydration markers delta-Ht, delta-BUN, delta-UA, and delta-Usg) or K (delta-K) were also plotted, and these correlations were assessed (Fig. 4). In the same way, the relation between Cr-eGFR and each hormonal value (FT3, free thyroxine (FT4), thyroid stimulating hormone (TSH), and insulin-like growth factor (IGF1)), or delta-Cr-eGFR was assessed; these changes (delta-FT3, delta-FT4, delta-TSH, and delta-IGF1) depending on BMI-SDS were also assessed (Fig. 5).

**Fig. 6:**
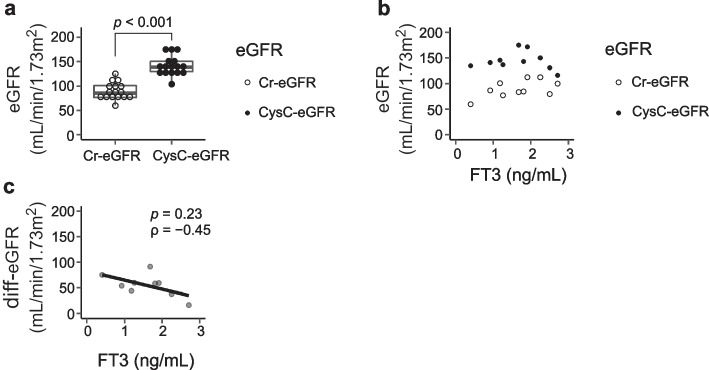
Differences between CysC-eGFR and Cr-eGFR depending on FT3. The dissociation between Cr-eGFR and CysC-eGFR evaluated simultaneously is shown. **a** CysC-eGFR exhibited a statistically higher value than Cr-eGFR (*p*  < 0.001). **b** In more than half of the cases, eGFR was assessed for FT3 < 2.0 ng/mL. **c** A tendency for a negative correlation between diff-eGFR and FT3 was implied, despite no statistical correlation being found (*p*  = 0.23, ρ  =  − 0.45)

To evaluate the difference between Cr-eGFR and CysC-eGFR, we next extracted the data for Cr-eGFR and CysC-eGFR evaluated at the same time, and a box plot was created. For these cases, Cr-eGFR and CysC-eGFR were evaluated several times, and the first evaluated values were adopted. We next extracted the data for simultaneously evaluated Cr, CysC and FT3, and created a scatter plot showing the relation between GFR estimated by Cr or CysC and FT3. Furthermore, we assessed the difference between CysC-eGFR and Cr-eGFR (diff-eGFR), and the correlation between diff-eGFR and FT3 was examined (Fig. 6).

### Statistical analysis

In the present study, the Wilcoxon signed-rank test was used to compare the data at the lowest and the highest BMI-SDS. We also compared the Cr-eGFR and CysC-eGFR data using the Mann–Whitney *U*-test. In our dataset, we determined that none of the parameters could be considered normally distributed, so we used non-parametric tests in the statistical analysis. Correlations between delta-Cr-eGFR and delta-BMI-SDS or changes in the other laboratory parameters were assessed using Spearman correlation coefficients. We defined a value of *p* < 0.05 as statistically significant. We used R commander (version 2.3–0) software based on R version 3.3.2.

## Results

### Patient characteristics

**Fig. 7:**
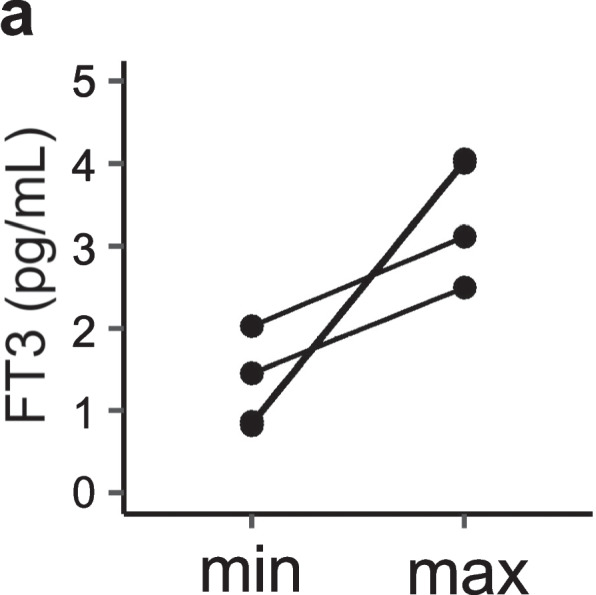
Changes in FT3 in cases in which FT3 was measured at both the lowest and highest points of BMI-SDS. In patients for whom FT3 was measured at both the lowest (min) and highest (max) points of BMI-SDS, FT3 recovered to the reference value in all instances

First, we examined the characteristics of our patients at the first evaluation of Cr-eGFR (Table 1) and at the points of the highest and lowest BMI-SDS (Table 2). Most data in Table 1 and the lowest BMI-SDS data in Table 2 were evaluated at approximately the same time. However, the evaluations at the time of Table 1 included many test items, such as FT3 and CysC, which were the main foci of this study. In this study, the data from 39 patients were evaluated; most of whom were pubertal girls with AN-R (Table 1). Of our 39 patients, 33 required hospitalization during the course of their illness. At this point, there was a general trend toward low HR and SBP. FT3 showed low values at the first evaluation of Cr-eGFR (Table 1), and similar features were demonstrated in the data during the lowest BMI-SDS (Table 2). Cr-eGFR at the lowest BMI-SDS was statistically lower than at the highest BMI-SDS (*p* < 0.001) (Table 2). Interestingly, the median value for CysC-eGFR (136.8 mL/min/1.73 m^2^) at the initial evaluation was much higher than that of Cr-eGFR (96.2 mL/min/1.73 m^2^), although CysC-eGFR was evaluated in some cases (Table 1). For Table 1, venous blood gas, skin turgor, and capillary refill time were each evaluated for only one patient, and all were judged to be normal. For the dehydration markers, statistical difference was found only for BUN (*p* < 0.001) between the lowest and highest BMI-SDS (Table 2). For the five patients for whom infusion therapy was initiated at the time of the lowest BMI-SDS and for whom “dehydration markers” were examined within 3 days, all “dehydration marker” values had improved. For patients for whom changes in Cr-eGFR were observed, Cr-eGFR values had also increased (Fig. 1). No statistical difference was found in K between the two conditions (Table 2). Although the level of FT3 increased at the point of the highest BMI-SDS in all cases, statistical analysis was not performed because of the small sample size (Table 2). The change in FT3 at the minimum and maximum BMI-SDS points are displayed and illustrated in Fig. 7.

Overall features are shown in Fig. 2 using all the data for all patients. A linear correlation was not found between Cr-eGFR and BMI-SDS (Fig. 2). Severe hypokalemia was not found, but dehydration was suspected in some patients with low BMI-SDS (Fig. 2). FT3 and IGF1 ameliorated along with the increase in BMI-SDS. (Fig. 2). Whereas the data shown in Fig. 2 shows the overall trends, the frequency of assessments widely differed depending on the respective patient. Therefore, we further examined the relation between Cr-eGFR and BMI-SDS or other parameters.

### Correlation between Cr-eGFR and BMI-SDS

As shown in Fig. 2, the analysis of all data suggested that there was no linear correlation between BMI-SDS and Cr-eGFR. However, it appeared that there might be a negative correlation when BMI-SDS was low and a positive correlation when BMI-SDS was high. Therefore, we created a LOESS curve and observed a negative correlation when BMI-SDS was ≤  − 4.5 and a positive correlation when BMI-SDS was >  − 1.5. We then sought to determine whether similar trends are present in individual cases.

Figure 3 shows the correlations between Cr-eGFR and BMI-SDS depending on each BMI-SDS category created along with the LOESS curve in scatter plots for Cr-eGFR and BMI-SDS, as shown in Fig. 2 (Fig. 3b, d, f). In addition to Cr-eGFR and BMI-SDS at the lowest and highest BMI-SDS values in each BMI-SDS category, the relation between delta-Cr-eGFR and delta-BMI-SDS is also shown (Fig. 3c, e, g). Despite the tendency for negative correlations with BMI-SDS ≤  − 4.5 SD and for positive correlations with − 4.5 < BMI-SDS ≤  − 1.5 SD or BMI-SDS >  − 1.5 SD that were implied between delta-Cr-eGFR and delta-BMI-SDS, no statistically significant correlation was found (Fig. 3b-g).

### Correlation between Cr-eGFR and dehydration markers or K depending on the change in BMI-SDS

We next examined the relation between Cr-eGFR and dehydration parameters or K, depending on the changes in BMI-SDS. Figure 4 showed statistically negative correlations between delta-Cr-eGFR and delta-BUN or delta-UA (Spearman correlation coefficient [ρ] =  − 0.42, *p* = 0.049 and *ρ* =  − 0.51, *p* = 0.014, respectively) (Fig. 4). No trend was found between (delta-)Cr-eGFR and the other parameters (Fig. 4).

### Correlation between Cr-eGFR and hormonal values depending on the change in BMI-SDS

Next, we examined the relation between changes in Cr-eGFR and each hormone depending on the change in BMI-SDS. Our data demonstrated a good correlation with delta-Cr-eGFR and delta-FT3 (*ρ* = 0.58, *p* = 0.0025), and the correlation between delta-Cr-eGFR and delta-TSH was also statistically significant (*ρ* = 0.36, *p* = 0.029) (Fig. 5b, f). There was no correlation between delta-Cr-eGFR and the other parameters, although the tendency for a positive correlation was implied between delta-Cr-eGFR and delta-IGF1 (Fig. 5h).

### Difference in the CysC-eGFR and Cr-eGFR of patients with AN

Because the data shown in Table 1 implied a dissociation between Cr-eGFR and CysC-eGFR, we next extracted the data for Cr-eGFR and CysC-eGFR evaluated at the same time and found that CysC-eGFR was statistically higher than Cr-eGFR (*p* < 0.001) (Fig. 6a). Scatter plots of eGFR and FT3 showed that more than half the eGFR data were evaluated during low FT3 (FT3 < 2.0 ng/mL) (Fig. 6b). We also studied the relation between FT3 and diff-eGFR. Although no statistical differences were found, a tendency toward a negative correlation was found between diff-eGFR and FT3 (Fig. 6c).

## Discussion

For our patients, changes in Cr-eGFR during treatment cannot be explained only by dehydration, chronic hypokalemia, or reduced muscle volume, even though dehydration partially influences renal function. Reduced thyroid function should be considered when evaluating the kidney function of patients with AN [15–17, 25].

To date, no study has described a detailed change in Cr-eGFR during the treatment course of AN. In our study, BMI-SDS followed a U-shaped or V-shaped curve in its association with Cr-eGFR (Figs. 1 and 7). This change cannot be explained only by muscle volume. It is known that reduced muscle volume decreases Cr, leading to apparently high Cr-eGFR, but actual changes in renal function depending on body weight can occur. Hyperfiltration in obese patients or decreased renal function in type 2 diabetes patients who have undergone a body weight loss of more than 10% has been documented previously [26, 27]. In animal studies, change in renal perfusion by weight gain or weight loss has been reported [28]. Thus, renal function can be affected by body weight itself in the case of AN, as well as by an apparent change in Cr by reduced muscle mass. For the patients of our study with severely reduced body weight (BMI-SDS ≤  − 4.5) we suspect that Cr-eGFR increased because of reduced muscle mass. However, transiently reduced Cr-eGFR became evident following an increase in body weight; one possible mechanism is a change in renal perfusion caused by a change in body weight.

The pathophysiology of decreased kidney function has been described for patients with AN [7, 8]. In such populations, intravascular volume depletion or chronic hypokalemia is thought to be the cause of decreased Cr-eGFR [7, 8]. At the start of treatment or at the lowest BMI-SDS, most patients did not show findings of hypokalemia (Tables 1, 2), and chronic hypokalemia was less likely to be the cause of decreased renal function in our patients (Figs. 2 and 4). Dehydration was assessed in our study using various parameters. Generally, the most reliable finding for dehydration is reduced body weight, but evaluating dehydration by body weight is difficult in patients with AN. The dehydration parameters used in this study are also useful for evaluating extracellular fluid volume, but sensitivity and specificity are not appropriate [29, 30]. The relative utility of BUN and UA in the assessment of dehydration has been reported in studies on dehydrated children [30]. Statistical correlations between Cr-eGFR and these values were found in our study (Fig. 4), however careful interpretation of these data is required because both BUN and UA are affected by dietary composition and because BUN is also affected by muscle catabolism. When examining changes in dehydration parameters before and after fluid infusion at the time of the lowest BMI-SDS (Fig. 1b-e), it was found that all dehydration marker values improved. Additionally, for patients in whom Cr-eGFR was examined, the Cr-eGFR level also increased (Fig. 1a). Considering these results, it seems that dehydration was at least partially the cause of the decreased Cr-eGFR in these patients and that the dehydration markers used in this study were reasonably useful. However, the Cr-eGFR level after fluid infusion (Fig. 1a) was somewhat low when considering the age-based reference values (mean value ± standard deviation: 115.2 ± 18.3 mL/min/1.73 m^2^) [23], leaving some doubt as to whether fluid infusion alone restored the Cr-eGFR to the level of these patients before illness. In our patients, dehydration may have had some effect on renal function, but the changes in Cr-eGFR during treatment could not be explained by dehydration alone.

Accurate evaluation of kidney function is difficult in patients with AN. Cr can be estimated as falsely low in these patients [31]. For patients with reduced muscle mass, CysC is known to be a useful marker [25] and it usefulness in AN patients has been documented [13]. However, there have also been some reports indicating that CysC-eGFR did not accurately reflect the GFR of patients with AN [31, 32]. In our study, dissociation between Cr-eGFR and CysC-eGFR was prominent. This dissociation (diff-eGFR) showed a negative correlation between FT3, with a relatively high Spearman correlation coefficient (*ρ* =  − 0.45), although statistical significance was not found (Fig. 4). In patients with hypothyroidism, dissociation between Cr-eGFR and CysC-eGFR has been described previously [33]. Low-FT3 syndrome is thought to act protectively for survival by lowering FT3 through the suppression of the hypothalamic–pituitary–thyroid axis activity during severe illness [34]. There are not many cases of chronic fatigue syndrome that meet the diagnostic criteria for low-FT3 syndrome [35]. Additionally, like hypothyroidism, low-FT3 syndrome is also suggested to potentially affect kidney function [36]. It was also reported that CysC-eGFR can be overestimated in patients with hypothyroidism because cellular turnover and metabolism are suppressed [36, 37]. In basic medical research, it has been demonstrated that T3 stimulates the production of cystatin-C in osteocytes and hepatocytes in a dose-dependent and time-dependent manner [38, 39]. Thus, it was estimated that what affects CysC is FT3 itself and the dissociation between the two parameters found in our patients was largely attributed to decreased FT3, as most of our patients showed low-FT3 syndrome during low BMI-SDS.

The real GFR level of patients with AN may decrease because of low-FT3 syndrome. In our study, a positive correlation between delta-FT3 and delta-Cr-eGFR was demonstrated (Fig. 3b). Similar outcomes were obtained in a previous study that reported improvement in renal function following treatment for hypothyroidism [16]. Other studies have shown the influence of hypothyroidism on renal function [15, 17, 40]. Studies have also reported a significant association between chronic kidney disease (CKD) and thyroid dysfunction or low-FT3 syndrome [41, 42]. In patients with CKD, the prevalence of thyroid dysfunction ranged from 48 to 58% [41]. Furthermore, decreased clearance of ^51^Cr-EDTA was shown in patients with severe hypothyroidism and this finding improved after hormone substitution therapy [17]. These data showed the effect of thyroid hormones on kidney function.

Disturbed renal function may be attributed to mechanisms provoked by hypothyroidism. Thyroid function plays an important role in regulating the renin–angiotensin–aldosterone system (RAAS) [43]. In fact, RAAS is activated by binding T3 to the nuclear β-adrenergic receptor and promoting renin gene expression [43]. Hypothyroidism can also disturb sodium and water reabsorption in the proximal tubule, and this activates tubule–glomerulus feedback, leading to reduced GFR [44–46]. Other effects of hypothyroidism, such as decreased synthesis of erythropoietin and red blood cells and impaired systolic and diastolic functions of the heart, may also affect GFR [47, 48]. A histological study in a hypothyroid rat model revealed changes such as atrophied glomeruli, mesangial cell proliferation, swollen capsular epithelium, and interstitial fibrosis [40]. Previously, some studies have indicated FT3 or TSH as a factors affecting renal function [49–51]. Even within normal reference ranges, changes in FT3 and TSH can affect the kidney function of patients for whom thyroid function is normal [52, 53]. Therefore, changes in TSH or FT3 may affect kidney function, either during hypothyroidism or even when thyroid function is normal. In our patients, FT3 had a stronger correlation with Cr-eGFR (Fig. 3b) and, considering the above reports, FT3 may have affected the Cr-eGFR of our patients with AN. Despite these changes being reversible in most cases of short periods of hypothyroidism [17], prolonged renal dysfunction might occur, especially in children [54]. In addition, our study suggested a correlation between TSH and Cr-eGFR, but this was weaker than the correlation between FT3 and Cr-eGFR. Similarly, the rate of change in Cr-eGFR was smaller relative to the rate of change in TSH. Furthermore, although our study showed a positive correlation between TSH and Cr-eGFR (Fig. 3f), many previous studies have reported a negative correlation between these two parameters [50, 51]. Therefore, our results require careful interpretation to rule out the possibility of chance.

Renal function might be affected by other hormones. The growth hormone/IGF1 axis is known to affect kidney function [55, 56], and IGF1 influences renal hemodynamics [55]. It has been demonstrated that IGF1 dilates mainly efferent arterioles, leading to increased renal flow [56]. Although no statistical relation was demonstrated in our study, a tendency toward a positive correlation between the delta-Cr-eGFR and delta-IGF1 levels was found (Fig. 3h). The serum IGF1 level can be influenced by thyroid dysfunction as well as low nutrition conditions [57]. Therefore, low-FT3 syndrome may affect Cr-eGFR by lowering the IGF1 level of patients with AN.

This study has some limitations. First, not all patients were in the incipient stage of AN because refractory cases were often introduced to us from other hospitals. Thus, the assessment of dehydration or serum potassium values may have been influenced by the treatment performed in these hospitals. Second, the number of patients was relatively small, thus we were not able to sufficiently evaluate some parameters. Third, because this was a retrospective study, treatment and laboratory tests varied by the attending physician, which may have affected the outcomes. Fourth, the relation between FT3 and Cr-eGFR, the divergence between CysC and Cr-eGFR, and their relation to FT3, as shown in this study, indicate correlations rather than direct causation. To prove a causal relation, many related factors need to be considered. However, multivariate analysis is challenging when evaluating highly correlated hormones, making it difficult to strictly prove causality with clinical data.

In conclusion, factors other than dehydration that affect renal function must be considered for patients with AN. Change in FT3 may be a useful factor, with renal function being suppressed by low-FT3 syndrome. Caution is required when interpreting CysC-eGFR in patients AN because CysC-eGFR may overestimate kidney function due to low-FT3 syndrome; evaluating renal function by Cr-eGFR may be preferred, but with the caveat that actual GFR may be lower than Cr-eGFR because of reduced muscle volume. This study suggests that many factors, including changes in FT3, dehydration, and/or reduced muscle mass, need to be considered when evaluating the kidney function of patients with AN; however, further research is needed to clarify these mechanisms.

## Data Availability

Data sharing is not applicable.

## Abbreviations

acWT: Actual weight
AN: Anorexia nervosa
AN-BP: Binge-eating/purging type of anorexia nervosa
AN-R: Restricting type of anorexia nervosa
BMI-SDS: Body mass index standard deviation score
BT: Body temperature
BUN: Blood urine nitrogen
CKD: Chronic kidney disease
Cr: Serum creatinine
Cr-eGFR: Glomerular filtration rate estimated by creatinine
CysC: Serum cystatin-C
CysC-eGFR: Glomerular filtration rate estimated by cystatin-C
DBP: Diastolic blood pressure
delta-BMI-SDS: Change of body mass index standard deviation score
delta-BUN: Change of blood urine nitrogen
delta-Cr-eGFR: Change of glomerular filtration rate estimated by creatinine
diff-eGFR: Difference between CysC-eGFR and Cr-eGFR
delta-FT3: Change of free triiodothyronine
delta-FT4: Change of free thyroxine
delta-Ht: Change of blood hematocrit
delta-IGF1: Change of insulin-like growth factor
delta-K: Change of potassium
delta-TSH: Change of thyroid stimulating hormone
delta-UA: Change of uric acid
delta-Usg: Change of urine specific gravity
eGFR: Estimated glomerular filtration rate
FBT: Family based treatment
FT3: Free triiodothyronine
FT4: Free thyroxine
GFR: Glomerular filtration rate
HR: Heart rate
Ht: Blood hematocrit
HT: Height
IGF1: Insulin-like growth factor
K: Potassium
LOESS: Locally estimated scatterplot smoothing
RAAS: Renin–angiotensin–aldosterone system
refCr: Reference serum creatinine
SBP: Systolic blood pressure
s-Cr: Serum creatinine
stWT: Standard weight
TSH: Thyroid stimulating hormone
UA: Uric acid
Usg: Urine specific gravity
