# Correction: Assessment of the renal function of patients with anorexia nervosa

**DOI:** 10.1186/s13030-024-00319-3

**Published:** 2024-10-21

**Authors:** Hiroyuki Miyahara, Yoshie Shigeyasu, Chikako Fujii, Chie Tanaka, Mana Hanzawa, Akiko Sugihara, Ayumi Okada, Hirokazu Tsukahara

**Affiliations:** 1 Department of Clinical Pediatrics, Okayama University Academic Field of Medicine, Dentistry, and Pharmaceutical Sciences, 2-5-1 Shikata-Cho, Kita-Ku, 700–8558 Okayama Japan; 2 Department of Pediatrics, Okayama University Hospital, 2-5-1 Shikata-Cho, Kita-Ku,, 700–8558 Okayama Japan; 3 Clinical Psychology Section, Department of Medical Support, Okayama University Hospital, 2-5-1, Shikata-Cho, Kita-Ku, 7008558 Okayama Japan; 4 Department of Pediatrics, Dentistry and Pharmaceutical Sciences, Okayama University Graduate School of Medicine, 2-5-1 Shikata-Cho, Kita-Ku, 700–8558 Okayama Japan

**Correction**: ***BioPsychoSocial Med*18**,** 19 (2024)**

10.1186/s13030-024-00316-6

Following publication of the original article [1], the authors reported that one author’s name Mana Hanzawa was in wrong display order as Hanzawa Mana. Thus, the name display order has been corrected.

The original article [1] has been updated.
